# Supplementary material for: Biological network growth in complex environments: A computational framework
Source: PLoS Comput Biol. 2020 Nov 30;16(11):e1008003. doi: 10.1371/journal.pcbi.1008003 (PMC7728203; doi:10.1371/journal.pcbi.1008003)
Supplement: S1 Text — (PDF) [file pcbi.1008003.s001.pdf]

# Efficient Computation of 3D Random Walks in Complex Environments using Structure Tensors and Angular Gaussian Distributions

This text contains supplementary information for the paper

**Biological Network Growth in Complex Environments: A Computational Framework**

by Torsten Johann Paul and Philip Kollmannsberger

*Center for Computational and Theoretical Biology, University of Würzburg*
*Campus Hubland Nord 32, Würzburg, Germany*

**3D RANDOM WALKS AND MULTIVARIATE GAUSSIAN DISTRIBUTIONS** ........ **1**

TRANSLATING SPATIOTEMPORAL SIGNALS INTO MULTIVARIATE GAUSSIAN DISTRIBUTIONS ........ 5
INTERNAL AND EXTERNAL DRIFT ........ 5
STRUCTURAL CONSTRAINTS ........ 7
*Obstacles & Surfaces* ........ *7*
*Attractive & repulsive obstacles/surfaces* ........ *9*
SPATIAL ORGANIZATION ........ 10
SUMMARY ON SPATIOTEMPORAL CUES & CONSTRAINTS ........ 11

**TRANSFORMATION OF THE MVG TO RESTRICTED GAGD** ........ **11**

COORDINATE TRANSFORMATION ........ 12
RESTRICTION OF THE FIELD OF VIEW ........ 13
GROWTH LENGTH AND ORIENTATION SEPARATION ........ 13
SUMMARY ........ 13

**COLLISION DETECTION** ........ **14**

# 3D Random Walks and Multivariate Gaussian Distributions

In the following text, we will further explain how the 3D random walk process underlying network development in a complex, time-variant environment can be approximated using Multivariate Gaussian Distributions. We start with Equation [1],

$$\frac{\delta p(\vec{x},t)}{\delta t} = \nabla\big(\vec{u}(\vec{x},t)p(\vec{x},t)\big) + \nabla(\mathrm{D}(\vec{x},t)\nabla p(\vec{x},t)) \qquad (1)$$

which is the so-called Fokker Planck equation. This equation describes the time development of a diffusive particle density, here expressed as a probability density function (pdf) $p(\vec{x},t)$ of a single particle that is under the influence of a drift $p(\vec{x},t)$ and diffusion $D(\vec{x},t)$. In general, these conditions are time- and space dependent.

The Fokker-Planck equation is the fundamental partial differential equation (pde) to describe multi-dimensional random walk processes in biology and commonly used in biophysics to describe stochastic motion and its statistical moments.

If $p(\vec{x}, t_n, \vec{u}, \boldsymbol{D})$ is a particle's spatio-temporal probability density function at time $t_n$, and if the random variable $\vec{X}$ is a point in space drawn out of the probability space $(\mathbb{R}^k, \mathcal{L}(\mathbb{R}^k), p(\vec{x}, t_n, \vec{u}, \boldsymbol{D}))$ with sample space $\mathbb{R}^k$, Lebesgue sigma algebra and probability function $p(\vec{x}, t, \vec{u}, \boldsymbol{D})$, then the integral

$$P(\vec{X} \in \mathrm{A}) = \int_A p(\vec{x}, t_n, \vec{u}, \boldsymbol{D}) dA$$

with $A = \int_{x_1}^{x_1+\epsilon_1} \dots \int_{x_k}^{x_k+\epsilon_k} dx_1 \dots dx_k$ calculates the probability to find a particle within this volume at time $t_n$.

In case of constant drift and diffusion, if the diffusion tensor is diagonal and isotropic and under the condition that the initial distribution of a particle at time $t_0$ is the delta distribution $p(\vec{x}, t_0) = \delta(\vec{x} - \vec{x}_0)$, the fundamental solution for equation [1] is equation [2]:

$$p(\vec{x}, t; \vec{u}, \boldsymbol{D}) = \frac{1}{\sqrt{(2\pi)^k (Dt)^k}} e^{\left(-\frac{(\vec{x}-\vec{x}_0-\vec{u}t)^2}{4Dt}\right)} \quad (2)$$

This equation is usually referred to as the fundamental solution and random kernel for stochastic motion and diffusive processes.

One can directly identify this probability density function with a Multivariate Gaussian function (MVG): the diffusion tensor is the covariance $\Sigma$ and the mean $\vec{u}t$ is the shift of the Gaussian probability density function. For the anisotropic diffusion tensor, the covariance is

$$\boldsymbol{D}t = \begin{pmatrix} 2D_1 & \cdots & 0 \\ \vdots & \ddots & \vdots \\ 0 & \cdots & 2D_k \end{pmatrix} t = \begin{pmatrix} \sigma_1^2 & \cdots & 0 \\ \vdots & \ddots & \vdots \\ 0 & \cdots & \sigma_k^2 \end{pmatrix} = \Sigma \quad (3)$$

with determinant $|\boldsymbol{D}t| = \prod_{i=1}^{k} 2\, D_i t$. Since $\vec{u}$ and **D** are constant, we can rewrite equation [2] as equation [4]:

$$p(\vec{x}, t; \vec{u}, \boldsymbol{D}) = \frac{1}{\sqrt{(2\pi)^k |Dt|}} e^{\left(-\frac{1}{2}\sum_{i=0}^{k} \frac{\left(x_i - x_{0,i} - u_i t\right)^2}{2D_i t}\right)} \quad (4)$$

As stated in the manuscript, analytical solutions for equation [1] can only be found for the condition that $\vec{u}$ and **D** are overall constant. If this is true for a time interval $\tilde{t} = t_0 + \delta t$ and

$t_0$ is the starting point with $p(\vec{x}, t_0) = \delta(\vec{x})$, we can split the process in $\tilde{t} = \sum_{i=0}^{n} dt = ndt$ substeps:

$$[t_0 < t_1 < \cdots < t_n = \tilde{t}, \qquad ndt = \tilde{t}]$$

With $\vec{X}_{dt}$ a random vector out of $P\big(\vec{X} \in \mathbb{R}^k, dt, \vec{u}, \boldsymbol{D}\big)$, we can build the iterative sum over n time steps $dt$ to generate a random vector $\vec{X}_{\tilde{t}} = \sum_{i=1}^{n} \vec{X}_{dt,i} = \vec{X}_{dt,1} + \cdots + \vec{X}_{dt,n}$ with the probability density functions

$$p(\vec{x}; \vec{u}, \boldsymbol{D}) = \frac{1}{\sqrt{(2\pi)^k |Ddt|}} e^{\left(-\frac{1}{2}\sum_{i=0}^{k} \frac{(x_i - u_i dt)^2}{2D_i dt}\right)} \tag{5}$$

for each $\vec{X}_{dt}$ in a series of time steps.

The probability density function for the summed random vector $\vec{X}_{\tilde{t}}$ can then directly be obtained by convolution of the individual pdfs:

$$p(\vec{x}, \tilde{t}; \vec{u}, \boldsymbol{D}) = p(\vec{x}; \vec{u}, \boldsymbol{D})_1 * \ldots * p(\vec{x}; \vec{u}, \boldsymbol{D})_n \tag{6}$$

Each pdf is a Multivariate Gaussian distribution of the same dimensions, therefore the convolution is calculated by the superposition of the covariances $\sum_{i=1}^{n} \boldsymbol{D}dt = \boldsymbol{D}ndt = \boldsymbol{D}\tilde{t}$ and the means $\sum_{i=1}^{n} \vec{u}dt = \vec{u}ndt = \vec{u}\tilde{t}$. This is one of the advantages of working with Gaussian functions for diffusive processes.

The splitting of the time interval is the numerical approach to simulate the time development of the diffusive processes. In each time step, a random vector is drawn from a pdf that is a Gaussian distribution. This description of diffusion is also called "Brownian Motion" or "Wiener Process with drift". Later on, we leave the picture of the "Wiener Process" when adding correlation and persistence to the process but for now, we have a discrete time series of random vectors that are Gaussian distributed and independent.

This convolution property and the superposition of covariances and means give the possibility to add different diffusive and drift cues originating from different signals during a single time step $t_i = idt$ at spatial position $\vec{x}_i$.

For the approach to describe the network growth model as diffusive process and to motivate this ansatz from a biophysical point of view, we make some assumptions on $\vec{u}(\vec{x}, t)$ and $\mathbf{D}(\vec{x}, t)$:

- First, we assume that the time development of $\vec{u}(\vec{x}, t)$ and $\mathrm{D}(\vec{x}, t)$ is constant or a very small $\varepsilon$ over some period of time $\tilde{t}$, $\frac{d}{dt}\vec{u}(\vec{x}, t) = \varepsilon$ and $\frac{d}{dt}\mathbf{D}(\vec{x}, t) = \begin{pmatrix} \varepsilon_1 & \cdots & 0 \\ \vdots & \ddots & \vdots \\ 0 & \cdots & \varepsilon_k \end{pmatrix}$.

- Second, we look at the spatial properties of $\vec{u}(\vec{x})$ and $\mathbf{D}(\vec{x})$ during the discrete time interval $\tilde{t}$. If vector $\vec{x}_i$ is the position of a diffusing particle during any time step $t_i$ in $\tilde{t} = \sum_{i=0}^{n} dt = ndt$ and $\nabla u(\vec{x}_i)t|_{t=t_i}$ and $\begin{pmatrix} \frac{\partial}{\partial \mathrm{x_i}} 2D_1(\vec{x}_i)t\Big|_{t=t_i} & \cdots & 0 \\ \vdots & \ddots & \vdots \\ 0 & \cdots & \frac{\partial}{\partial \mathrm{x_k}} 2D_k(\vec{x}_i)t\Big|_{t=t_i} \end{pmatrix}$ are continuous under the discrete differential operator $\frac{\partial}{\partial x} f(x) \approx \frac{f(x+\varepsilon)-f(x)}{\varepsilon}$, then the pdf of a random vector $\vec{X}_{dt,i}$ could depend on space as follows:

$$p(\vec{x}; \vec{u}(\vec{x}_i), \boldsymbol{D}(\vec{x}_i))_i = \frac{1}{\sqrt{(2\pi)^k |\boldsymbol{D}(\vec{x}_i)dt|}} e^{\left(-\frac{1}{2}\sum_{j=0}^{k} \frac{\left(x_j - u_j(\vec{x}_i)dt\right)^2}{2D_j(\vec{x}_i)dt}\right)} \quad \textbf{(7)}$$

We split the time series $\sum_{i=0}^{n} dt$ into steps n of length $dt$. From now on, we use $\vec{\mu}$ for the mean and the symbol $\boldsymbol{\Sigma}$ for the covariances or diffusion tensor, and allow the tensors to be anisotropic:

$$p(\vec{x}; \vec{\mu}(\vec{x}_i), \Sigma(\vec{x}_i))_i = \frac{1}{\sqrt{(2\pi)^k |\boldsymbol{\Sigma}(\vec{x}_i)|}} e^{\left(-\frac{(\vec{x}-\vec{\mu}(\vec{x}_i))^T \boldsymbol{\Sigma}(\vec{x}_i)^{-1} (\vec{x}-\vec{\mu}(\vec{x}_i))}{2}\right)} \quad \textbf{(8)}$$

This leads to the following pseudo code for the numerical diffusive motion:

$$\vec{x}_p = \vec{x}_{init}$$

for i in iterations($t_0 < t_1 < \cdots < t_n = \tilde{t}$):

$$\vec{x}_p = \vec{x}_p + \vec{f}(\vec{X}_{dt,i} \in P_{conv}(\vec{x}; \vec{\mu}_{conv}(\vec{x}_p), \boldsymbol{\Sigma_{conv}}(\vec{x}_p)))$$

$$P_{conv} = \overbrace{\int_A p_{conv}\left(\vec{x}; \vec{\mu}_{conv}(\vec{x}_p), \boldsymbol{\Sigma_{conv}}(\vec{x}_p)\right) dA}$$

$$p_{conv} = \overbrace{p_1(\vec{x}; \vec{\mu}_1(\vec{x}_p), \boldsymbol{\Sigma_1}(\vec{x}_p)) * \ldots * p_c(\vec{x}; \vec{\mu}_c(\vec{x}_p), \boldsymbol{\Sigma_c}(\vec{x}_p))}$$

**Table 1: Pseudo code for the growth process, in each time step $t_i$ a new random step vector $\vec{f}$ is added to the current position. $\vec{f}$ is a function depending on the pdf combining all cues and constrains.**

The function $\vec{f}$ operates on the random vector $\vec{X}_{dt,i}$ and its pdf. This random vector is drawn from the pdf $p_{conv}\left(\vec{x}; \vec{u}_{conv}(\vec{x}_p), \boldsymbol{\Sigma_{conv}}(\vec{x}_p)\right)$. Furthermore, is $\vec{f}$ a function that deals with the shortcomings of the MV Gaussian kernel to describe stochastic motion in a physically meaningful way: in general, a MVG covers the full $\mathbb{R}^k$, which could lead to unphysical growth

velocities $\vec{v} = \frac{\vec{X}_{dt,i}}{dt}$ for $\vec{X}_{dt,i} \to \infty$ if $\vec{X}$ is interpreted as the end position of a growth step during the time interval $dt$.

The pdf $p_{conv}\left(\vec{x}; \vec{\mu}_{conv}(\vec{x}_p), \boldsymbol{\Sigma}_{conv}(\vec{x}_p)\right)$ that occurs in the discrete growth sequence

$$\vec{x}(t_{i+1}) = \vec{x}(t_i) + \vec{f}(\vec{X}_{dt,i} \in P_{conv}(\vec{x}; \vec{\mu}_{conv}(\vec{x}(t_i)), \boldsymbol{\Sigma}_{conv}(\vec{x}(t_i)))) \tag{9}$$

is calculated by the convolution of all pdfs that act as constraints on the moving particle. In general, after the convolution the pdf would look like

$$p_{conv} = p_{iso} * p_{drift,1} * \dots * p_{drift,n} * \dots * p_{diffuion} * p_{substrate} * p_{persistence} * p_{cue,1} * \dots * p_{cue,n} \tag{10}$$

In the next section, we discuss how different cues and constraints are transformed into MVGs and how the function $\vec{f}$ is defined.

## Translating spatiotemporal signals into Multivariate Gaussian Distributions

We interpret all occurring internal and external signaling cues as well as structural constraints acting on the growth direction as individual Multivariate Gaussians. Each MVG distribution takes 9 parameters: the covariance $\Sigma = (\sigma^2_{ij})_{i,j \in [x,y,z]}$ describing the shape and metric of the MV Gaussian has 6 parameters (since the matrix is symmetric), and the mean $\vec{\mu}$ shifting the center of the distribution away from the center of the Cartesian coordinate system is a 3-dimensional vector. For each individual signal, the mean $\vec{\mu}$ indicates the most likely direction a growth cone will grow towards. Its length indicates the strength of the signal, while the covariance describes the signal shape:

$$p(\vec{x}; \vec{\mu}, \Sigma) = \frac{1}{\sqrt{(2\pi)^3|\Sigma|}} e^{\left(-\frac{(\vec{x}-\vec{\mu})^T\Sigma^{-1}(\vec{x}-\vec{\mu})}{2}\right)}, \qquad \vec{\mu} = \begin{pmatrix} \mu_x \\ \mu_y \\ \mu_z \end{pmatrix}, \boldsymbol{\Sigma} = \begin{pmatrix} \sigma_{xx}^2 & \sigma_{xy}^2 & \sigma_{xz}^2 \\ \sigma_{xy}^2 & \sigma_{yy}^2 & \sigma_{yz}^2 \\ \sigma_{xz}^2 & \sigma_{yz}^2 & \sigma_{zz}^2 \end{pmatrix}$$

Note that $\vec{\mu}$ does not encode a signal's position in space (its origin in the computation field), but $\vec{\mu}(\vec{x}_p)$ is a vector that points in direction $\vec{\mu} \sim \frac{\vec{x}_s - \vec{x}_p}{\|\vec{x}_s - \vec{x}_p\|}$ if $\vec{x}_p$ and $\vec{x}_s$ are the particle's and signal's position in the computation field. The length of the vector $\|\vec{\mu}\|$ is the strength the signal has at position $\vec{x}_p$. One could say that a particle is "pulled" in direction $\vec{x}_s - \vec{x}_p$.

## Internal and external drift

In this section, we describe some internal as well as external drift cues that could be present during the growth process. In general, a drift towards a specific direction is expressed as a MVG with $\vec{\mu} = a\vec{e}_x + b\vec{e}_y + c\vec{e}_z$ and $\Sigma$ with $\sigma_{ij} \xrightarrow{\text{for i=j}} 0$, else 0. This way the MVG distribution

tends towards the 3D delta distribution $\delta(\vec{x}-\vec{\mu})$ but is still a MVG with all its useful properties.

- A constant drift induced at each local growth cone position $\vec{x}_p$ could be the result of a signal at position $\vec{x}_s$ with $||\vec{x}_p|| << ||\vec{x}_s||$.
- Locally changing drift forces could be transmitted via a three (x,y,z) layered gradient field or a vector field where the source is the field that pulls the cone in direction of the local gradient / vector with magnitude that is the vector length / gradient slope.

The schematic below (Image 1a) sketches the 3 layers of the computation field containing the 3 components of the drift field. Here, the drift is constant, therefore $c\vec{e}_z = (0,0,0)^T, b\vec{e}_y = (0,-.5,0)^T, a\vec{e}_x = (1,0,0)^T$, but in general, we have structured layer volumes with locally varying drift magnitudes and orientations. The second image (Image 1b) shows the drift represented as MVG distribution for any position in the constant drift computation field.

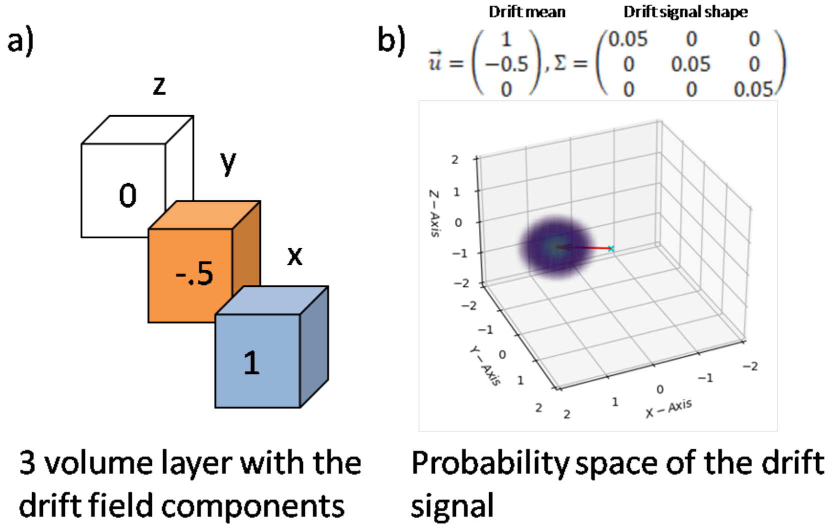


Image 1: a) Constant drift field, each position in the computation field yields a constant value to pull the growth cone in direction (1,-.5,0). b) Representation of the drift field as MVG with σ² = 0.005.

- An example for internal drift that is not induced via a local gradient is the correlation with previous growth steps. In this case, $\vec{\mu}$ would be the normalized vector of the n last growth steps $\vec{\mu}_{mem} = \frac{\sum_{i=0}^{n} \vec{d}_{m-i}}{\|\sum_{i=0}^{n} \vec{d}_{m-i}\|}$ added. The corresponding $\Sigma$ with $\sigma_{ij} \xrightarrow{\text{for i=j}} 0$, else $0$ would again be a very sharp peak. This contribution to the cone movement correlates the next likely orientation with the previous orientations. As every drift property is a MVG, they add up under convolution. The images below show a sequence of growth steps in 2D where the mean vector of the MVG distribution that encodes the correlation is the normalized sum of the n steps before.
- The movement could also be internally biased. Different types of network edges (‘phenotypes’) could likewise tend to move in distinguished directions. This again would result in a sharp MVG always pointing in the direction $\vec{\mu} \sim \vec{x}_{bias}$.

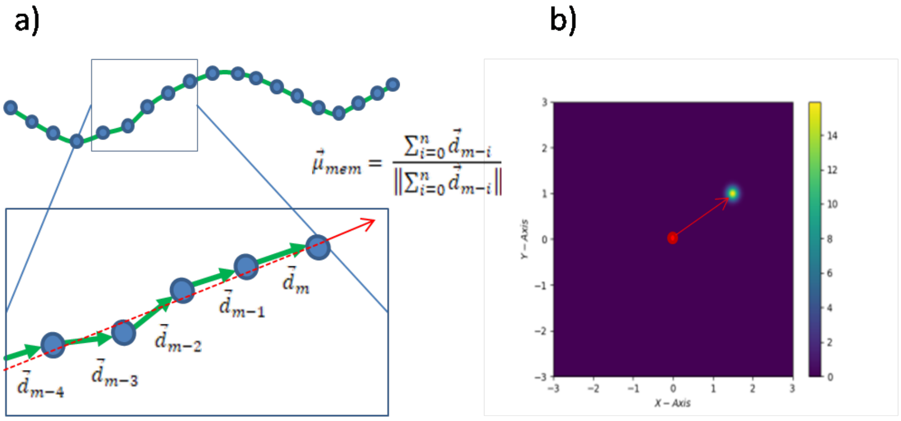


Image 2: a) Correlation (persistence) of the following growth step with the steps before b) 2D MVG of the correlation drift

## Structural constraints

External and internal structural constraints are handled in the same manner, but the covariance as the metric of the MVG distribution now carries the local structure information. It holds information about the axes’ scaling and how the principle axes of the MVG are rotated relative to the coordinate system of the growth process.

## Obstacles & Surfaces

The structure tensor has the same symmetric form as the diffusion tensor of the anisotropic random motion. This property is used in image processing for anisotropic diffusion filtering, where each voxel in an image is Gaussian filtered with respect to the structural surroundings. The application of an anisotropic Gaussian filter process (a time series of blurring with increasing $\sigma_i^2$) that is constrained by edges (regions with significant gradients) matches the kernel of our random motion process. Therefore we use this to represent the structure of the substrate in which a growth cone is embedded as a MVG. In the following images, we show as example a computation field that contains a wall. This field is decomposed into its spatial structural components, the structure tensor and its derivatives.

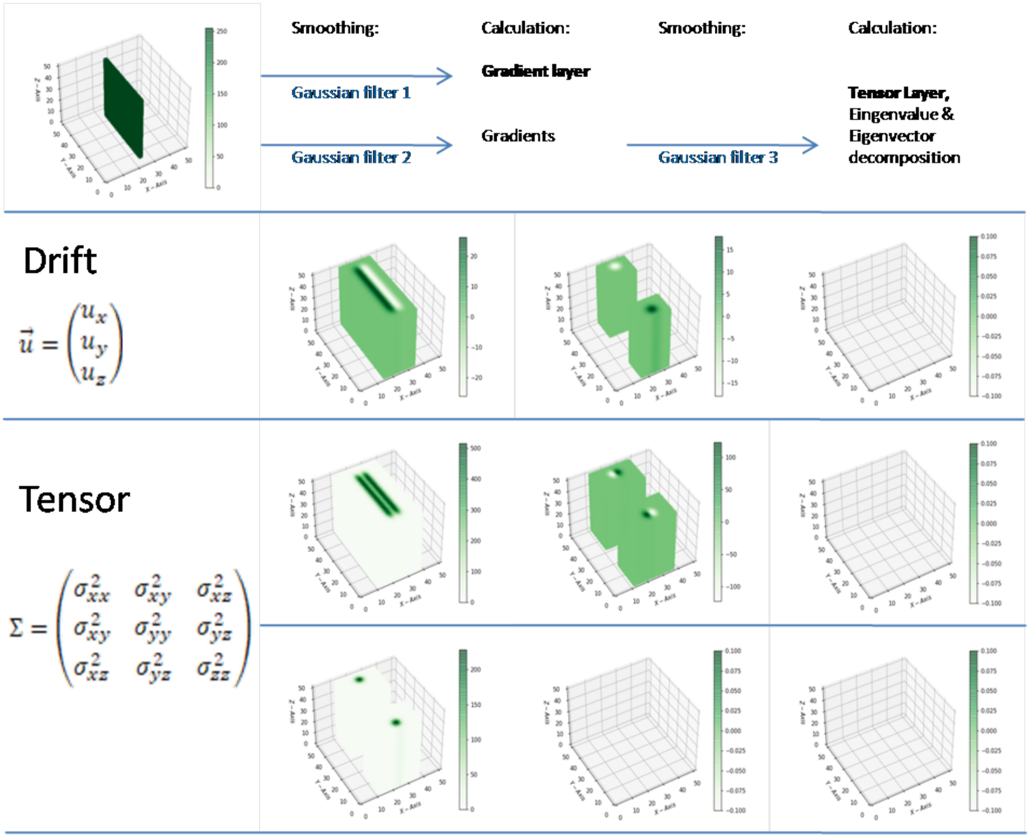


**Image 3: The image shows the layer for the nine MVG parameters that describe a wall obstacle. The image containing the obstacle is Gaussian filtered in each step. This blurring creates a "nearfield".**

Each voxel in the computation field is now represented by its initial value and 9 features. The analysis of the eigenvalues shows that for a wall, we generate $\lambda_1 \approx \lambda_2 \ll \lambda_3$ eigenvalues. A MVG that would contain such a metric as covariance would look like a tube or extremely elongated cigar. Therefore, we have to use the dual space (the inverted axes or reciprocal space) of the structure tensor to substitute it with the diffusion tensor in the MVG:

- Step 1: Decomposition of the structure tensor into eigenvalues and eigenvectors

$$\mathbf{\Sigma}_{\text{diag}} = \begin{pmatrix} \lambda_1 & & \\ & \lambda_2 & \\ & & \lambda_3 \end{pmatrix}, A = (\vec{v}_1 \quad \vec{v}_2 \quad \vec{v}_3)\ ,\ \mathbf{\Sigma} = A^T \mathbf{\Sigma}_{\text{diag}}\, A$$

- Step 2: Transformation to inverted eigenvalues

$$\begin{aligned} \vec{a}_x &= \vec{e}_x \lambda_1 \\ \vec{a}_y &= \vec{e}_y \lambda_2 \\ \vec{a}_z &= \vec{e}_z \lambda_3 \end{aligned} \qquad \begin{aligned} \vec{a}_{x,d} &= \frac{\vec{a}_y \times \vec{a}_z}{<\vec{a}_x, \vec{a}_y \times \vec{a}_z>} \\ \vec{a}_{y,d} &= \frac{\vec{a}_z \times \vec{a}_x}{<\vec{a}_y, \vec{a}_z \times \vec{a}_x>} \\ \vec{a}_{z,d} &= \frac{\vec{a}_x \times \vec{a}_y}{<\vec{a}_z, \vec{a}_x \times \vec{a}_y>} \end{aligned}$$

- Step 3: rescaling of the inverted eigenvalues from reciprocal length & backtransformation

$$\lambda_{1,d}^{sc} = \lambda_{1,d}\frac{\max(\Sigma_{\mathrm{diag}})}{\max(\Sigma_{\mathrm{diag}}^{\mathrm{dual}})}$$

$$\lambda_{2,d}^{sc} = \lambda_{2,d}\frac{\max(\Sigma_{\mathrm{diag}})}{\max(\Sigma_{\mathrm{diag}}^{\mathrm{dual}})} \qquad \boldsymbol{\Sigma}_{\mathrm{diag}}^{\mathrm{dual,sc}} = \begin{pmatrix} \lambda_{1,d}^{sc} & & \\ & \lambda_{2,d}^{sc} & \\ & & \lambda_{3,d}^{sc} \end{pmatrix} \qquad \boldsymbol{\Sigma}^{dual,sc} = A^T \boldsymbol{\Sigma}_{\mathrm{diag}}^{\mathrm{dual,sc}} A$$

$$\lambda_{3,d}^{sc} = \lambda_{3,d}\frac{\max(\Sigma_{\mathrm{diag}}^{\mathrm{dual}})}{\max(\Sigma_{\mathrm{diag}}^{\mathrm{dual}})}$$

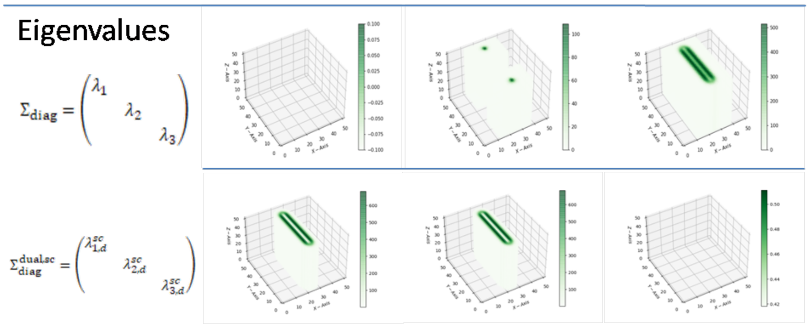


**Image 4: Eigenvalues after the structure tensor's eigendecomposition and eigenvalues transformed and rescaled in dual space**

After transforming the eigenvalues and basis into dual space, they are rescaled. This way, we achieve a length inversion multiplied by the quotient of the largest eigenvalue and the largest eigenvalue of the dual space. The largest eigenvalue is associated to the eigenvector that is the normal to the surface or tangent plane of an obstacle or structure. The transformation into dual space will thus generate two large eigenvalues that span this plane and that are of the largest eigenvalue's magnitude. This way, a growing edge or moving particle will follow a random orientation that with the highest probability is parallel to the surface's tangent plane.

## Attractive & repulsive obstacles/surfaces

We can give those MVGs with transformed covariance an additional mean vector to describe if the structure is repulsive or attractive. For example, the random growth along a surface has a covariance with two eigenvalues and corresponding eigenvectors spanning the tangent plane of the surface curvature. The third eigenvector points in normal direction but its eigenvalue is near zero. The membrane could for example be the wall in the image above. The drift points in negative normal direction to hold the moving particle on the surface. The strength of the drift is obtained from them membrane's gradient field. One further necessity is the application of a Gaussian or other smoothing filter by which the multilayer computation grid is blurred in order to obtain continuous fields (that decrease with distance). The use of Gaussian filters also generates an "effective nearfield" of the structures. A particle moving towards an obstacle will see increasing mean $||\vec{\mu}||$ and $\boldsymbol{\Sigma}$ eigenvalues. The image below shows the MVG associated to a voxel that is in the "near field" of a horizontal wall or membrane.

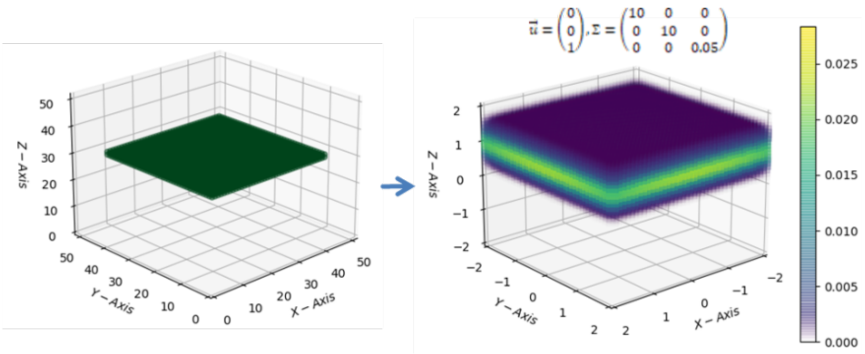


**Image 5: MVG for one voxel near the wall obstacle whose drift feature in z direction yields 1**

Blurring an image is equivalent to reducing its resolution, thus we have a further degree of freedom to change the resolution and therefore the size of the individual grid layers. This justifies our use of the term "images" for the layers: we not only apply classical image analysis tools such as gradient filters to computation fields, but one can directly use images obtained from microscopes that contain obstacles such as cell bodies as computation fields. If we use volume images as computation fields in which growth cones are moving, we filter the volume with the structure tensor filter and get 9 layers: three for the gradients and 6 for the tensor parameters. We thus need 9 parameters to describe every voxel of a 3D substrate by a MVG.

## Spatial organization

Apart from obstacles and surfaces, the substrate can also be characterized by an intrinsic organization. For example, intercellular space could contain an underlying extracellular matrix such as collagen fibers. These matrices can again be described by their structure tensor. In the image below, a 2D filament matrix spans the volume. This net has two principal axes rotated relative to the Cartesian coordinate frame with different eigenvalues. If this anisotropy of space is expressed as a tensor and used as covariance in a MVG, it results in a non-shifted, anisotropic random walk reflecting the properties of the matrix.

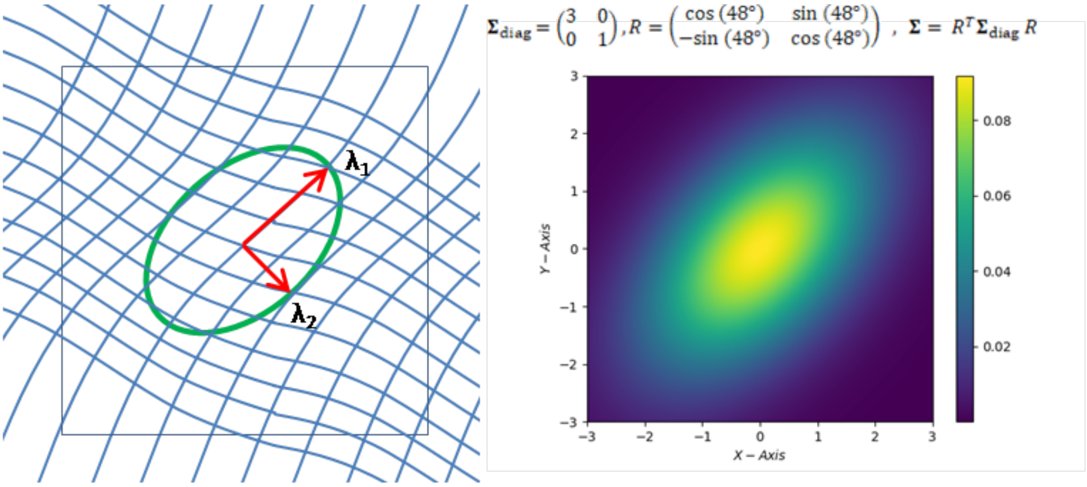


**Image 6: Spatial organization of a 2D filament net that structures the space and defines the preferred orientations**

## Summary on spatiotemporal cues & constraints

Taken together, each growth cone collects an ensemble of MVGs at its position, where each MVG represents either a signaling cue, spatial constraint, a drift force, a substrate structure metric and so on. Each individual MVG would require drawing a random number for the most likely random vector anisotropically distributed around its mean. Instead of generating each of these random vectors individually, we can combine the MVG pdfs into a common pdf via convolution, and as all pdfs are Gaussian, the resulting pdf is Gaussian, too. Biologically, one could compare this to the picture that the cones as agents possess sensory organs which "filter" their surroundings accordingly for cues and relevant signals. Each sensor presents a probability distribution to decide where to move next, and the agent combines them into a single probability distribution that stochastically determines the next direction.

# Transformation of the MVG to restricted GAGD

The sum of two random numbers/vectors $\vec{a}$ , $\vec{b}$ is a random number/vector whose pdf is the convolution of the pdfs of a and b. Since the convolution of two Gaussians is again a Gaussian distribution, the convolution of two Gaussian pdfs is also a Gaussian pdf. These 3D MVG distributions cover the full $\mathbb{R}^3$ and have a mean $\vec{\mu}_{conv} = \sum_{i=1}^{n} \vec{\mu}_i$ that is the sum of all $m$ means and a covariance $\Sigma_{conv} = \sum_{i=1}^{n} \Sigma_i$ that is the sum of all $m$ covariances. A random vector $\vec{X}$ drawn from a 3 dimensional MVG without any further restricting conditions has a length distribution between 0 and $\infty$ and an orientation distribution between 0 to $4\pi$ with $\vec{X}(R, \Phi, \Theta) = R(cos(\Phi)\sin(\Theta), \sin(\Phi)\sin(\Theta), \cos(\Theta))^T$.
From a biophysical and simulation perspective, this is problematic: if each growth step happens within a time interval $dt$, random vectors with extreme magnitude would produce unphysical growth speed distributions. To draw a random vector out of $\mathbb{R}^3$, one needs to sample the full space in a meaningful and computationally efficient way. A sampling of the pdf to a subset of $\mathbb{R}^3$ can solve this problem but needs to conserve the pdf's general properties defined by its covariance and mean.

To solve this problem, we define a function $\vec{f}(\vec{X}_{dt,i} \in P_{conv}(\vec{x}; \vec{\mu}_{conv}(\vec{x}(t_i)), \boldsymbol{\Sigma_{conv}}(\vec{x}(t_i))))$ that acts upon the pdf, as illustrated in Image 7.

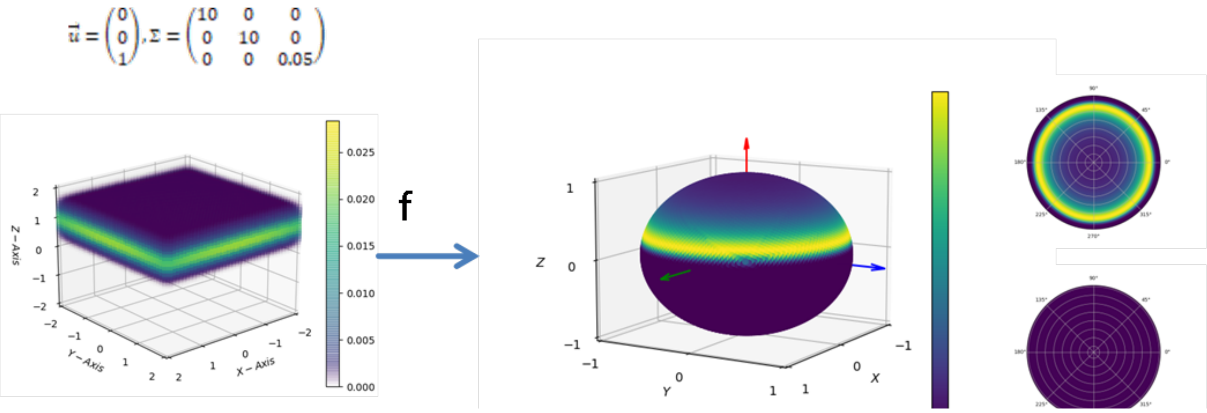


**Image 7: The transformation of a MVG into a general angular Gaussian distribution (GAGD) is given via the function f. This function integrates the MVG over its radial component. The angular MVG distributes random unit vectors on the unit sphere $S^2$ according to its mean and covariance.**

$\vec{f}$ Is a function that transforms Cartesian coordinates to spherical coordinates and afterwards integrates $p_{angular} = \int_{r=0}^{\infty} p\left(r, \varphi, \theta; \vec{u}(\vec{x}_i), \Sigma(\vec{x}_i)\right)_i dr$ to obtain a distribution on the sphere, as shown in Image 7: on the right side, the spherical distribution is together with polar plots of both hemispheres (north and south). This transformation fulfills all requirements mentioned above and elegantly maintains the picture of the agents' "sensor array" observing its horizon. To draw the next random orientation, we sample a certain number of points on the sphere using an equidistant mapping based on entangled Fibonacci series, and run a Monte Carlo Simulation.

## Coordinate transformation

The coordinate transformation in its general formulation reads

$$\vec{x} = r\vec{y}, \vec{y} = \frac{\vec{x}}{\overrightarrow{\|\vec{x}\|}}, r = \|\vec{x}\| \quad (11)$$

such that a infinitesimal volume element is defined by

$$\vec{x} = r^{k-1} d\vec{y}, \text{ for} \in \mathbb{R}^k \quad (12)$$

In 3 dimensions, this is expressed in spherical coordinates $(r, \varphi, \theta)$ as

$$\begin{pmatrix} x \\ y \\ z \end{pmatrix} = r \begin{pmatrix} \cos(\varphi)\sin(\theta) \\ \sin(\varphi)\sin(\theta) \\ \cos(\theta) \end{pmatrix}, dxdydz = r^2 \sin(\theta) dr d\theta d\varphi \quad (13)$$

The integration over the radial component

$$GAGD(\varphi, \theta, \vec{\mu}, \mathbf{\Sigma}) = \int_{r=0}^{\infty} MVG(r, \varphi, \theta, \vec{\mu}, \mathbf{\Sigma},) r^2 \sin(\theta) dr d\theta d\varphi \quad (14)$$

results in the following distribution

$$GAGD(\vec{y}, \vec{\mu}, \mathbf{\Sigma}) = \frac{1}{\sqrt{(2\pi)^2 |\mathbf{\Sigma}|}} \frac{1}{\sqrt{\vec{y}^T \mathbf{\Sigma}^{-1} \vec{y}}} e^{\left[\frac{1}{2}\left(\frac{\left(\vec{y}^T \mathbf{\Sigma}^{-1} \vec{\mu}\right)^2}{\vec{y}^T \mathbf{\Sigma}^{-1} \vec{y}} - \vec{\mu}^T \mathbf{\Sigma}^{-1} \vec{\mu}\right)\right]} * M_2\left(\sqrt{\frac{(\vec{y}^T \mathbf{\Sigma}^{-1} \vec{\mu})^2}{\vec{y}^T \mathbf{\Sigma}^{-1} \vec{y}}}\right) \quad (15)$$

with the factor $M_2(\alpha)$ defined as

$$M_2(\alpha) = \int_{u=0}^{\infty} u^2 \frac{1}{\sqrt{2\pi}} e^{\left(-\frac{(u-\alpha)^2}{2}\right)} du, \alpha = \frac{\vec{y}^T \mathbf{\Sigma}^{-1} \vec{\mu}}{\sqrt{\vec{y}^T \mathbf{\Sigma}^{-1} \vec{y}}} \quad (16)$$

## Restriction of the field of view

We further restrict the agent’s observed area on the sphere surface to a solid angle $\Omega(\tilde{\theta}_{app}, \varphi_{rot}, \theta_{rot})$, where $\tilde{\theta}_{app}$ is the half aperture angle opening a spherical cap from the north pole. The area of this surface is then rotated relative to the north pole axis in direction $\vec{r}(\varphi_{rot}, \theta_{rot})$, as illustrated in Image 8. This restriction results in self-avoidance within the persistence length, the Kuhn length.

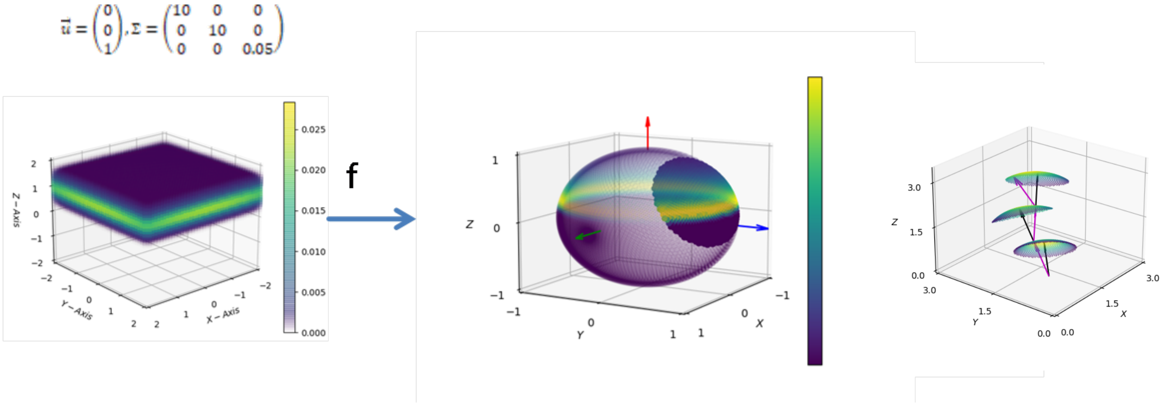


**Image 8: Random orientations are restricted to the surface area inside the solid angle $\Omega(\tilde{\theta}_{app}, \varphi_{rot}, \theta_{rot})$. Right: Three consecutive movement steps, magenta shows the drawn orientation and the arrows in black are the rotated north pole of the solid angle. The rotation maps the north pole to the orientation of the late step.**

## Growth length and orientation separation

The last functionality of $\vec{f}$ is the separation of the distributions of length and orientation. While $GAGD(\varphi, \theta; \vec{\mu}(\vec{x}_i), \Sigma(\vec{x}_i))_i = \int_{r=0}^{\infty} MVG(r, \varphi, \theta; \vec{\mu}(\vec{x}_i), \Sigma(\vec{x}_i))_i dr$ is solely a distribution to draw a random orientation, one is free to multiply these vectors with a scalar to obtain variable step lengths. This scalar could be drawn from an independent step length distribution. In the implementation and the framework, we use a constant step length of 1.

## Summary

Our framework uses a multilayer “image” grid for the computation field. These layers represent the external cues and constraints. Each cue and constraint has nine layers corresponding to its nine parameters, and each layer is a three dimensional volume providing a parameter value for each position $\vec{x}_p$ of an agent. These layers can vary in terms of resolution and complexity, e.g. an overall constant drift force at each poinit $\vec{x}_p$ just needs nine layers that are each 1x1x1 voxels in size, or several cues and constraints could be combined into “shared” layers, or all obstacles including walls, cell bodies, impassable terrain, attractive surfaces and so on can be stacked into the nine layers describing the substrate topology.
The movement of the agents themselves is gridless, and their position is a floating-point number. All cues and constraints present at $\vec{x}_p$ are summed up to generate a MVG to draw the next direction, thereby reducing the dimensionality from $(x, y, z) \xrightarrow{transform} (r, \varphi, \theta)$ to $(\varphi, \theta)$ by integrating over the radial component.

## Collision detection

To check for collisions between edges and growth cones, there is a hidden grid layer ("flavor field") that stores the IDs of the agents. Its resolution equals that of the highest resolved "image" layer. This layer does not change the agents' floating-point position but stores their ID at the nearest grid point. By using the hidden "flavor"-layer to compute collisions, we reduce checks for interactions from complexity $O(N^2)$ to $O(N)$.
