# Supplementary material for: Biological network growth in complex environments: A computational framework
Source: PLoS Comput Biol. 2020 Nov 30;16(11):e1008003. doi: 10.1371/journal.pcbi.1008003 (PMC7728203; doi:10.1371/journal.pcbi.1008003)
Supplement: S1 Table — (PDF) [file pcbi.1008003.s002.pdf]

**External Cues**	$\vec{\mu}$	Σ	$\Sigma_{diag}(\lambda_1,\lambda_2,\lambda_3)$
Signals:			
-Drift Signal	$\sim \alpha(\vec{x}_p)\frac{\vec{x}_s-\vec{x}_p}{\lvert\vec{x}_s-\vec{x}_p\rvert}$	$\Sigma \sim \mathbb{I}$	$\lambda_i \to 0$
-const. Drift	$\vec{\mu} \sim \alpha\left(\frac{\vec{x}_s}{\lvert\vec{x}_s\rvert}\right)$	$\Sigma \sim \mathbb{I}$	$\lambda_i \to 0$
	$\lvert\vec{x}_p\rvert << \lvert\vec{x}_s\rvert$		
-Drift field (img)	$\nabla p(\vec{x}_p)$	$\Sigma \sim \mathbb{I}$	$\lambda_i \to 0$
**Internal Cues**	$\vec{\mu}$	Σ	$\Sigma_{diag}(\lambda_1,\lambda_2,\lambda_3)$
Correlation:	last m orientations:		
-Persistence &	$\frac{\sum_{i=0}^{m}\vec{r}(\phi,\theta)_{p-i}}{m}$	$\Sigma \sim \mathbb{I}$	$\lambda_i \to 0$
-Selfavoidance	$\Omega(\phi,\theta) : \vec{X}_\omega \in P(\vec{X} \in \mathbb{S}^2_{\Omega(\phi,\theta)}, \vec{\mu}_{conv}, \Sigma_{conv})$		
Internal Bias	$\vec{\mu}_{bias}$	$\Sigma \sim \mathbb{I}$	$\lambda_i \to 0$
**External Constrains**	$\vec{\mu}$	Σ	
Structure tensors:		Scaled Dual Tensor:	Transformation:
-attractive surfaces	$\nabla p(\vec{x}_p)$	$\Sigma_{ij}^{dual,sc} = \sigma_{ij}^2$	$\Sigma_{i,j}^{dual,sc} = R^T c \Sigma_{diag}^{dual} R$
-Obstacles	$-\nabla p(\vec{x}_p)$	Image Tensor:	$\Sigma_{diag} = R \Sigma_{i,j} R^T$
-spatial organization	$\vec{\mu} = \vec{0}$	$\Sigma_{ij} = \delta_i p \delta_j p$	$c = \frac{\lambda_1}{\lambda_3^{dual}}$
(metric)			
**Internal Constrains**	$\vec{\mu}$	Σ	$\Sigma_{diag}(\lambda_1,\lambda_2,\lambda_3)$
Stochasitc motion:			
-simple RW-Walk	$\vec{\mu} = \vec{0}$	$\Sigma \sim \mathbb{I}$	$\lambda_1 = \lambda_2 = \lambda_3$
-anisotropic RW-Walk	$\vec{\mu} = \vec{0}$	$\Sigma_{i,j} = \sigma_{ij}^2$	$\lambda_1 \geq \lambda_2 \geq \lambda_3$
