# Supplementary material for: Biological network growth in complex environments: A computational framework
Source: PLoS Comput Biol. 2020 Nov 30;16(11):e1008003. doi: 10.1371/journal.pcbi.1008003 (PMC7728203; doi:10.1371/journal.pcbi.1008003)
Supplement: S2 Table — (PDF) [file pcbi.1008003.s003.pdf]

Model	Space	Energy	Mechanism	Cues	Code available	Application
**This work**	**Hybrid 3D**	no	**Edges grow, branch, merge**	Arbitrary signals, structures on grid and anisotropy	**Python**	Generic
Cohen D. (1967)	Continuous 2D	no	Edges grow, branch, but do not merge	attractive/repulsive signals	no	Plants
Kaiser M, Hilgetag CC., (2004)	Continuous 2D	no	Edges form as straight lines between pre-existing nodes	no	no	Generic
**NETMORPH**<br>Koene RA, Tijms B, et al. (2009)	Continuous 2D/3D	no	**Edges grow, branch, merge**	no	**C++**	Neurons
**TREES**<br>Cuntz H, Forstner F, et al., (2010)	Continuous 3D	no	Edges form between existing nodes	Node density	**MATLAB**	Neurons
**CX3D**<br>Zubler F, Douglas, R, (2009)	**Hybrid 3D**	**yes**	**Edges grow, branch, merge**, nodes defined by triangulation	Diffusing signals, mechanical forces, fields	**Java**	Generic
Vanherpe L ,et al. (2016)	Continuous 3D	no	Edges grow and branch, but do not merge	Anisotropy, explicit domain boundaries	no	Neurons
Ronellenfitsch H, Katifori E., (2016)	Grid 2D	**yes**	Pruning of edges in regular “noisy” hexagonal network	no	no	Vessels
Hannezo E, Scheele CLGJ et al. (2017)	Continuous 3D	no	Edges grow and branch, but do not merge	Chemical gradients, anisotropy, boundaries	no	Generic
Perfahl H, Hughes BD et al. (2017)	Continuous 3D	no	Edges grow and branch, but do not merge	Chemical gradients, mechanical forces	no	Vessels
