# Supplementary material for: Biological network growth in complex environments: A computational framework
Source: PLoS Comput Biol. 2020 Nov 30;16(11):e1008003. doi: 10.1371/journal.pcbi.1008003 (PMC7728203; doi:10.1371/journal.pcbi.1008003)
Supplement: Attachment — Submitted filename: Response_to_Reviewers.pdf [file pcbi.1008003.s005.pdf]

Response to Referee Reports

We thank all reviewers for their detailed comments and for taking the time to read our work. Based on their helpful feedback, we prepared a revised and improved version of our manuscript.

The three main improvements are 1) a more detailed application to biological questions including quantitative comparisons, parameter studies, and reproducible code; 2) better explanations of the underlying concepts and assumptions, both in the manuscript and as supplementary text and code, and 3) a better discussion of differences and improvements in comparison to previous work.

Below, we provide a detailed list of our responses to the review comments (blue) and a description of the changes we have made in the manuscript (red).

Reviewer #1 (Reproducibility Report)

R1-Q1: Unable to reproduce the results associated with the computational implementation of the novel modeling approach presented in this manuscript. However, we were successful in obtaining the results of the test problem provided in the associated Google Colab demonstration. Model source codes, documentation and example configuration of the computational model developed are freely accessible under a MIT License and can be downloaded from Github via a link provided. These were used in assessing the reproducibilty of the work presented in this manuscript. The Google Colab implementation of the modeling approach successfully runs to produce a network growing on the surface of a sphere, as documented in the Github repository.

However, there was no documentation providing instructions on how to use the source codes provided to reproduce the results of the implementation shown and described in Figure 6.

Response: Thank you for testing the reproducibility of our model, and for the opportunity to take part in the pilot phase of this great initiative. We now provide a Jupyter notebook that reproduces the results shown in the new Figure 6. In addition, we now also provide a recipe file to build a Singularity container as another route for reproducible use of our code, as well as the script for the Slurm scheduler to run simulations in a HPC environment.

Changes to the manuscript: Additional Jupyter notebook, Singularity recipe and runscript used to generate the results shown in Fig. 6. The original Colab notebook was retained to still be able to quickly test our model without requiring local setup.

Reviewer #2

This manuscript addresses a major gap in our understanding of complex life: exploring generative processes which give rise to multicellular assemblies. It is very relevant work to an under-researched area. An agent-based model is presented which gives rise to complex branching structures. The model is well described. The outputs of this generative process appear to be interesting and potentially relevant.

R2-Q1: The biological relevance of the generative model could be made more explicit. In particular, how the various parameters in the model might be implemented or represented in a biological system. This would help support the inclusion of these parameters and being tangible modulators, rather than a series of variables capable of reproducing biological observations, but detached from how these processes actually occur.

Response: This is a very important point; we agree that our original text focused too much on the physical background rather than the underlying biological mechanisms. We now motivate better the choice of the parameters and to which biological process they refer by adding additional text and references to biological examples, and by complementing the list of parameters in Table 2 with their biological meaning. This also helps to assess the relevance of the parameter studies we now performed for the new Figure 6, as detailed below in our response to your questions R2-Q2 and R2-Q3.

Changes to the manuscript: Additional text and references to the biological meaning of the model parameters were added to the paragraph outlining the growth process (line 54 ff.) as well as to Table 2.

The paragraph describing the “Growth Process” (line 54 ff.) now reads:

„Edges turn into trees if branching and/or bifurcations occur*, such as in growing neurons, osteocytes or sprouting angiogenesis.* Finally, if edges can join other edges and form new nodes *(e.g. synapses)*, a connected network emerges. “

The paragraph describing the “Implementation” (line 209 ff) now reads:

„Besides these image data and the seed points and seed orientation, the simulation requires a number of model parameters such as step length, aperture and degree of persistence as input, listed in Table 2. *The aperture is the opening angle of the “field of view” of the growth cone. Smaller values limit the possible directions it can turn to, resulting in self-avoidance as described e.g. for neurons [44]. The angles for branching and bifurcations depend on the underlying biological mechanism, with large angles e.g. for vessel sprouting [46] or small angles for actin-crosslinker mediated branching of cell processes [47]. The corresponding probabilities for branching and bifurcation describe how likely these processes occur in a growing edge, which in biological systems can be both intrinsically determined (e.g. typical rates in osteocyte processes [14]) or externally controlled as in neurite outgrowth [2]. The parameter “memory” sets the number of previous steps that contribute to the next growth direction and determines the persistence of the direction. Biologically, persistent motion can arise either from physical constraints (e.g. persistence length of microtubules) or from the intrinsic persistence of the biological process, e.g. in cell migration. All parameters are set at the beginning of the simulation but can change for each growth cone as a function of time or external signals to capture the corresponding biological mechanism in the simulation.*

Table 2 was modified to include an additional column with information on how the respective parameter might be implemented or represented in a biological system.

R2-Q2: The networks generated by the model visually appear similar to the 2 biological networks they are compared to in the study. However, no quantitative comparison is provided. Using a variety of centralities to quantitatively compare the generative models to biological data would strengthen the conclusion that these models are capable of creating biologically meaningful outputs

Response: Thank you for bringing up this very important point. We now enhanced the manuscript by a detailed quantitative comparison, as also requested by reviewers #2 and #3. We understand that a more quantitative comparison is required to support the conclusion that our framework can create meaningful biological outputs. Our objective is to show that our model does not only reproduce local structure of networks, but global emergent topological parameters. For this, we also included centralities such as suggested by reviewer 2.

Changes to the manuscript: We now perform a quantitative comparison of model-generated networks with experimentally measured networks using topological properties and centralities and replaced Figure 6 with a new Figure showing the results. The corresponding text in the results section was completely rewritten to reflect the contents of the new figure.

The sentence in the abstract referring to the application now reads:

"…we perform growth simulations of *a dense network between cells and compare the results to experimental data from osteocyte networks in bone."*

R2-Q3: Following on from the previous point, a sensitivity analysis as to how the various parameters in the model impact the topological properties of the outputs from the model would be very useful. Again, using quantitative approaches via centrality measures, the range of topologies generated by the model, and the presence of critical points in parameters can be identified.

Response: Following this suggestion, we now present a sensitivity analysis for the parameters of the model as an additional table showing in which direction each parameter influences the global topological properties, such as centralities. By looking at the time development of the average node degree, we identified a critical point where the giant component emerges during the growth of a multicellular network, shown in the new Figure 6a.

Changes to the manuscript: We performed systematic parameter studies shown in the new Figure 6 and Table 4, including a sensitivity analysis of the global network topology to the local model parameters.

The corresponding text in the results section now describes also the sensitivity analysis:

Line 301: "…the results of the sensitivity analysis of global network topology on the growth parameters are shown in Table 4."

Reviewer #3:

I enjoyed reading this paper, and I believe the premise of the paper - a generalized model for biological network growth under a variety of conditions - is extremely promising. The authors put great thought and effort into deriving the mathematical principles of network growth (in particular, I thought it was very clever to model individual environmental cues as multivariate Gaussians that can be combined through convolution into a single aggregate "environment" constraint). I am by no means an expert in directional statistics or geometry (and I have some points of confusion described below) but from what I understood, the mathematical foundations of their network growth model appear sound.

R3-Q1: While the methods presented in this paper are exciting, I have concerns about publishing this without more empirical results. The authors present two anecdotal examples of using their network growth model to generate networks similar to those observed in biology. These two examples are not very convincing; in Figure 6, it is not obvious to me that the simulated network actually replicated the original network. And even if it did, these are just two examples. The authors are advertising a mathematical framework with a robust ability to generate a wide variety of biological networks by simply tweaking the input parameters, but they don't provide very much evidence of this. I realize this is easier said than done, but I think the paper would be strengthened with quantitative evidence that their network growth model can reproduce the growth seen in a variety of systems. This would likely involve finding a particular set of parameters that, when used in simulations, generates a sample of random

networks that are then shown to be quantitatively similar to observed networks from the same system (and then repeating this process for other systems).

Response: We completely agree that the initial version of our manuscript did not show the true capabilities of our framework for quantitative modeling, as was also noted by reviewers #2 and #4. We now performed a much more systematic and quantitative study including parameter variations, generated samples of networks with identical parameters, and directly compared the resulting properties with experimental data. Since the quantitative experimental study of 3D biological networks is an emerging field and data are still rare, we use the osteocyte lacuno-canalicular network as example, since rich 3D data are available.

Changes to the manuscript: We added a systematic quantitative comparison of simulated networks to experimental observations and replaced Figure 6 with a new figure. We also now generate a larger sample of random networks with identical starting parameters for statistical analysis. For details and the accompanying changes to the results, please see our response to R2-Q2 and R2-Q3 of Reviewer #2.

R3-Q2: typo: "our tool allows to study..." should this be "allows us to study..."? Note that this phrasing occurs throughout the paper, and if it is changed here it should be changed throughout.

Response: Indeed, thank you for spotting this typical "Germanism". We changed all occurrences either to "allows us", as suggested, or to "makes it possible to" where appropriate.

Changes to the manuscript: All occurrences of "allows to…" have been changed to "allows us to…" or to "makes it possible to".

R3-Q3: line 51-52: "making too many assumptions about these cues", and line 270-271: "not modelled explicitly, e.g. in terms of hard-coded conditions, but through a generic mechanism based on angular probability density functions." I think the authors would do well to more explicitly explain how their model for directional cues differs from prior work.

Response: Thank you for pointing this out, we now include a more detailed explanation how the approach for directional cues in our model differs from prior work. The key difference is that external constraints are modeled as a discrete grid derived from "image volumes" as shown in Figure 3, which makes it possible to use actual experimentally derived images, e.g. of the tissue environment in which the network develops. We explicitly compare this to previous work in the discussion and the newly added Supplementary Table 1, as also detailed in our response to question R4-Q9 by reviewer #4 below.

Changes to the manuscript: A new Supplementary Table 1 was added containing a detailed comparison of different model properties, including directional cues, with previously published work. We also extended the passages mentioned above in the reviewer comment to be more explicit in explaining how our model differs from prior work.

Changes to the manuscript: The paragraph in the discussion comparing our framework to prior work now reads:

Line 330: *"The first agent-based computer simulation of 2D spatial network growth, published in 1967 [20], already used a similar approach as our model: edges grow and branch according to rules, respond to attractive and repulsive cues, and follow density gradients. The next growth direction is randomly assigned based on sampling the space around the growth tip. Inspired by plant growth, this model can generate a variety of tree-like networks – a remarkable achievement given the limitations of computer simulations more than 50 years*

ago. While the field of complex networks flourished decades later, relatively little work was done on spatial network growth [1, 19]. In 2009, two new computational frameworks for neuronal network growth were published, CX3D [25] and NETMORPH [26]. Both use biologically realistic growing and branching rules and are openly available. In both models, synapse formation only takes place after the growth phase. CX3D offers a more physically realistic treatment of mechanical forces compared to our model, but lacks the flexibility of grid-based external structural cues. Two other neuron growth models [23, 53] efficiently generate neuron tree morphologies and space-filling networks, but without biologically realistic growth mechanisms and synapse formation. Taylor-King et al. [54] use an elegant mean field approach for network growth based on local state degree distributions. Whenever it is biologically plausible to analytically formulate local rules, such an approach can accurately reproduce global properties. In their model for vessel tree development, Perfahl et al. [55] include physical interactions with the environment and between edges to study the role of mechanical forces for vessel development. In their "Unifying theory of branching morphogenesis", Hannezo et al. [28] present a stochastic model for epithelial duct development based on branching and annihilating random walks. Although interactions are limited to explicitly defined chemical gradients and anisotropy, this model can reproduce experimental tree topologies for a variety of organs. We summarized this comparison of our model with prior work in a Table. To our knowledge, our work presents the first framework for network growth that includes a biologically motivated local growth process as well as arbitrary interactions with an external environment, and is fully available as computational implementation."

R3-Q4: are equations (1) and (2) necessary? As the authors state, an analytic solution is elusive, and the authors end up resorting to a numerical solution based on random walks.

Response: Although equations (1) and (2) end up not being used directly in our numerical implementation, they are nevertheless important to understand why we can use Multivariate Gaussians to model the stochastic random walk process underlying network formation: equation (1) makes the link to the biophysics of random walk processes, as it is the partial differential equation describing a single particle under the influence of drift and diffusion. We identify its analytical solution for constant drift and diffusion, equation (2) with a Multivariate Gaussian function and discretize it for time- and space-dependent external influences (drift and diffusion). This forms the basis of our numerical approximation.

This important connection was not well enough described in the previous version of the manuscript. We rephrased it for clarity, and additionally refer to a much more detailed explanation which we provide as new supplementary material.

Changes to the manuscript: Additional explanations have been added as supplementary text. The manuscript text around equations (1) and (2) has been rephrased and now reads:

Line 65: "In *general*, such a stochastic motion under the influence of external forces is described by *a partial differential equation (PDE),* the Fokker-Planck equation corresponding to the Langevin equation:"

Line 74: "Equation (2) describes the time development of a random movement for constant drift and isotropic diffusion *starting at initial position* x_0."

Line 69: "…k-dimensional diffusion tensor. *This equation describes the time development of a probability density function p(x,t) of diffusing particles under the influence of a drift u(x,t) and diffusion D(x,t).* If drift and diffusion are *both* constant…"

Line 80: ““For constant drift and diffusion (Equation 2), the corresponding PDF, or growth kernel, is a Multivariate Gaussian distribution (MGD) *with mean vector μ=…, covariance Σ=… and determinant **D***. In the general case, *assuming that the spatio-temporal change for μ and Σ is small between two consecutive time steps*, every discrete growth step has a different time- and position dependent PDF. *The fundamental solutions Equation 2 of the PDE (Equation 1), or Equation 3 for non-diagonal covariance, can thus be used as random kernels in each step from which the subsequent growth directions are drawn. Their μ and Σ can be expanded so that they encode for all spatial signals and constraints. A more detailed derivation of this approach starting from Equation (1) is provided as Supplementary Text S1.”*

R3-Q5: I'm having some trouble understanding lines 82-90. In particular, how should I interpret the "mean vector" \mu? Does this encode the location of some signal? Or does it encode the direction in which the current edge is being "pulled"? If it's the former, I think this needs to be better explained. If it's the latter, I believe it would be helpful to include a subscript, i.e. \mu_t, to indicate that this is a time-dependent value. This confusion carries on throughout the paper, and I think it could use a more intuitive explanation in lines 82-90.

Response: The mean $\vec{\mu}$ of the Multivariate Gaussian does not encode a signal’s position in space (its origin in the computation field), but $\vec{\mu}(\vec{x}_p)$ is a vector that points in direction $\vec{x}_s - \vec{x}_p$ if $\vec{x}_p$ and $\vec{x}_s$ are the particle’s and signal’s position in the computation field. The length of the vector $||\vec{\mu}||$ is the strength the signal has at position $\vec{x}_p$. It could indeed be said that a particle is being “pulled” in direction $\vec{x}_s - \vec{x}_p$.

Thank you for pointing this out, we rewrote the paragraph line 82-90, and refer to a more detailed explanation in the newly added supplementary text. We do not think it would help to add a subscript for time dependence, since it can be both time- and position dependent and is determined separately for each individual time step and signal. Instead, the new supplementary text contains pseudo code for a growth step series, and the discretization of $\vec{\mu}$ and therefore its time dependence is demonstrated.

Changes to the manuscript: We added some clarifications to the paragraph lines 82-90 (now 91ff.) and additionally refer now to the new supplementary text.

Line 91: “The individual PDFs that describe all the different internal and *external constraints and cues that act on an edge at position x_p* are formulated using the Multivariate Gaussian Distribution (MGD) as kernel”

Line 100: “Depending on the edge's current position x_p, the mean vector μ points to the most likely direction *the edge will move towards in the subsequent step,* while the covariance Σ reflects *the impact of the local structure onto its movement* in the complex environment.”

R3-Q6: line 96 - should "determinate" be "determinant"?

Response: Yes, “determinant” is the right mathematical term, thank you. We fixed this typo.

Changes to the manuscript: “determinate” was changed to “determinant” in the description following equation (3).

R3-Q7: Why does the document intermittently stop listing line numbers?

Response: Thank you for pointing out this error, which was due to missing gaps in the Latex source. It has been corrected in the revised manuscript.

Changes to the manuscript: Missing line numbers have been fixed.

R3-Q8: I am slightly confused about the section on projecting onto the unit sphere. My intuition is that we essentially generate a random direction vector, and then project it onto a sphere of a randomly chosen radius? Is this correct? If so, I think the paper would benefit from explaining this explicitly.

Response: We agree that this requires a more precise explanation and justification. The "projection" onto a sphere results from integrating over the radial component of the 3D Multivariate Gaussian distribution in equation (3), which covers the full R^3. Sampling the entire space would not only be prohibitively slow but would also generate unphysical step size distributions. Our solution to integrate over the radius in equation (7) generates a sampling of the PDF from a subset of R^3 while retaining the full spatial information contained in its covariance and mean. An additional advantage is the separation in two probability distributions for the two independent variables step size and growth direction, which can now be treated individually. To make this clearer, we added a passage stating the problems occurring with an unrestricted MVG kernel to motivate why we integrate over the radius. For further details, we added a reference to additional explanations in the new supplementary text.

Changes to the manuscript: We modified the section on projecting onto the unit sphere and now also refer to additional explanations in the new supplementary text.

Line 133, the first paragraph to the section "Step Length" now reads: "*The Multivariate Gaussian Distribution in Equation (3) covers the full R^3, but sampling the entire space to draw the next growth direction would not only be prohibitively slow, but would also generate unphysical step size distributions. This problem can be solved by integrating over the radius to generate a sampling of the PDF from a subset of R^3 (the unit sphere) while retaining the full spatial information contained in its covariance and mean. An additional advantage is that growth direction and step length are separated into two probability distributions which can then be treated independently.*"

Line 159, we added: "*For additional detail, please see Supplementary Text S1*."

R3-Q9: the MGD in equation (3) takes three inputs; but the MGD inside the integral in equation (7) takes only two inputs; why the disparity?

Response: We originally omitted the input parameters $\vec{\mu}$ and Σ in equation (7) to emphasize the coordinate transformation and the integration over the radial component. Actually, $\vec{\mu}$ and Σ are still present in $MGD(r, \vec{y})$ and in $MGD_{\text{angular}}(\vec{y})$, as can be seen in equation (8). To avoid confusion, we changed equation (7) to show all input parameters.

Changes to the manuscript: We added the missing inputs to equation (7), which now reads

$$MGD_{angular}(\vec{y}; \vec{\mu}, \Sigma) = \int_{r=0}^{\infty} MGD(r, \vec{y}; \vec{\mu}, \Sigma)\, r^{k-1} dr$$

R3-Q10: line 162: should there be seed directions, in addition to seed locations?

Response: Yes, thank you for noting this – it should be "seed locations and directions" since each seed point has three spatial coordinates (x,y,z) and two angles, as shown in Table 2. This has been corrected in the text and is now more explicitly stated in Table 2.

Changes to the manuscript: We changed "seed points" to "seed locations and directions" and added descriptions to the seed parameters at the end of Table 2.

Line 219 now reads: "Besides these image data and the seed *locations and directions*, …"

R3-Q11: I could not understand the purpose of representing the environment as a grid. Is this simply for clustering to efficiently compute collision and merge events? Otherwise, I am not sure what difference this makes since the coordinates of edges are still continuous.

Response: The grid representation of the environment in an otherwise continuous model, as illustrated in Figure 1, serves two purposes: 1) arbitrary external boundary conditions can be included without explicitly stating their geometry by including them as scalar data on a 3D grid or "image" layer, and 2) the complexity of collision detection with nearby edges is reduced by storing agent IDs at the nearest grid point in an additional hidden layer and checking if they are within the field of view of a growing cone. We added some explanations to the text to help to understand why we chose this hybrid discrete-continuous approach for our model.

Changes to the manuscript: We added a brief explanation to the paragraph describing the implementation:

Line 215ff. now reads: "This *grid-based representation of the environment in an otherwise continuous model, as illustrated in Figure 1, makes it possible* to study network growth in real tissue environments as measured e.g. with confocal or light sheet microscopy *without explicitly stating their geometry by including them as scalar data on a 3D grid or "image" layer".*

Line 249ff. now reads: "For merging, the cone *at position p* performs a collision detection with all edges of an ensemble … *that are within its search field of radius R.Ω* includes all edges…"

Line 253: "…*that fulfils the condition to be in shortest distance to the cone position*."

R3-Q12: Figure 6: are the blue/green square drawing, and the blue circular drawing, both graphical representations of the same simulation? This was confusing to me

Response: They were from the same simulation, but different cells were mixed up in the blue circular network representation. Figure 6 in the original manuscript has been replaced by a new Figure – for details, please see our responses to your first question above and to Reviewer #2.

Changes to the manuscript: We replaced Figure 6.

Reviewer #4:

The authors developed a computational model of biological network growth in complex environment. A computational framework is based on directional statistics to model network formation in space and time under arbitrary spatial constraints. Growth is described as a biased correlated random walk where direction and branching depend on the local environmental conditions and constraints. To demonstrate the application of the computational model, authors performed growth simulations of the osteocyte lacuno-canalicular system in bone and of the zebrafish sensory nervous system.

It does a nice job of introducing relevance and background for such a question and overall I think it is interesting computational material. Unfortunately, a couple of things have put me a bit off enjoying the quality and impact of the paper's results:

R4-Q1: The structure of extracellular space is named “images”. What is description of “images”? A more detailed description of the «image» concept must be given.

Response: We understand that in the initial version of the manuscript, we did not sufficiently describe the concept of the grid- or image-based representation. We use the term “grid” and “image” interchangeably, since the representation of the underlying data is identical: a three-dimensional array of scalar values. While the positions of growth cones, nodes and edges are stored as continuous floating-point values, the external cues are stored as a discrete grid or image volume, as illustrated in Figure 1.

We use the term “images” because one can use real experimentally obtained image volumes, e.g. of tissue environments in which the network grows, and compute gradient features using image filters as shown in Figure 3 to generate the computing grid with the external cues. We added some explanations to the text to help to understand why we chose a hybrid discrete-continuous approach for our model and what we mean by “images” here. For further details, please also refer to our response to question R3-Q11 by reviewer #3.

Changes to the manuscript: We replaced “images” by “grid” in the abstract for better clarity and added a description of the “image” concept to Line 215 ff (see response to question R3-Q11 by reviewer #3).

R4-Q2: The variable p is introduced in the equation 1, but it is not written what this variable means in the framework of the model.

Response: Thank you for pointing out this missing information. The variable p is the probability density function $p(\vec{x},t)$ of a single particle that is under the influence of a drift $p(\vec{x},t)$ and diffusion $D(\vec{x},t)$. In the initial version of the manuscript, the link between equations (1) and (2) for a general random walk process, and our approach to solve these equations numerically for arbitrary external signals was not clear – this is now fixed: we now provide a detailed explanation as supplementary material and refer to it in the manuscript. The short description in the manuscript was rephrased for clarity. For details, please also see our response to the corresponding questions R3-Q4 by Reviewer #3.

Changes to the manuscript: Additional explanations have been added as supplementary text. The manuscript text around equations (1) and (2) has been rephrased and now reads:

Line 65: “In *general*, such a stochastic motion under the influence of external forces is described by *a partial differential equation (PDE),* the Fokker-Planck equation corresponding to the Langevin equation:”

Line 74: “Equation (2) describes the time development of a random movement for constant drift and isotropic diffusion *starting at initial position* x_0.”

Line 69: “…k x k-dimensional diffusion tensor. *This equation describes the time development of a probability density function p(x,t) of diffusing particles under the influence of a drift u(x,t) and diffusion D(x,t).* If drift and diffusion are *both* constant…”

Line 88: “*A more detailed derivation of our approach starting from equation (1) is provided as Supplementary Text S1.”*

R4-Q3: On page 5 (line 78) the vector u is indicated in bold, although throughout the entire article vectors are indicated by an arrow at the top. It is necessary to use the same designations throughout the article.

Response: Thank you for pointing out the inconsistent notation in line 78. This has been fixed, we now use arrow notation for vectors and bold for tensors, such as the diffusion tensor $\boldsymbol{D}$, throughout the entire article.

Changes to the manuscript: Page 5, Line 78, $\boldsymbol{u}$ has been replaced by $\vec{u}$.

R4-Q4: It is necessary to describe possible biophysical mechanisms of "self-avoidance", and references to experimental works must be provided.

Response: We now offer a better biological description for the different parameters and processes, including self-avoidance, in our model. This is related to the first question R2-Q1 by reviewer #2. We added a reference to experimental work to the paragraph on "Self-Avoidance" and a brief explanation to the "Aperture" parameter in Table 2.

Changes to the manuscript:

Line 188, Section on "Self-Avoidance": "Local self-avoidance *is the phenomenon that growing edges in a biological network, e.g. outgrowing neurites or branching vessels, do not loop back onto themselves (ref). In our growth model, local self-avoidance* is achieved…"

R4-Q5: What does means parameter "memory" in Table 2? There is no specific description or explanation in the text. Does this mean computational memory?

Response: We apologize for the confusion – no, it is not computational memory, but biological "memory" and/or persistence of the growth direction. The numerical value of this parameter defines the number of previous directions to which the next growth direction is correlated. We extended Table 2 to provide a biological meaning of the parameters, including memory – please see also our response to question R2-Q1 by reviewer #2.

Changes to the manuscript: Table 2 has been modified to include a brief explanation of all parameters, including "memory". The section on "Implementation" now contains biological interpretations of the different model parameters, including "memory":

Line 228: "*The parameter "memory" sets the number of previous steps that contribute to the next growth direction and determines the persistence of the direction. Biologically, persistent motion can arise either from physical constraints (e.g. persistence length of microtubules) or from the intrinsic persistence of the biological process, e.g. in cell migration*."

R4-Q6: The model does not take into account energy consumption of growing branch at all. The authors should to explain in the text of the article, why this important process (which can create restrictions) does not included into the model.

Response: This is a very good point; we did not justify why our model does not include energy consumption, although it is a key parameter in biological processes. The reason is that it would not only require an "energy" parameter for each growth cone (which would be trivial to implement), but also to explicitly model different possible mechanisms of energy sources, energy distribution and consumption. For example, energy could either be "harvested" from the environment by the growing processes or distributed throughout the network from cell bodies, depending on the specific type of network to be modeled. In order to keep our model as generic as possible, we decided to omit energy consumption, but due to the openly available code, it can be added by anyone who is interested in modeling a specific system. We now mention this limitation in the discussion section and include energy consumption in

our comparison of our framework with previous work, as detailed in our response to your question R4-Q9 below.

Changes to the manuscript: We added a short paragraph about energy consumption to the discussion of the limitations of our model. We also mention energy consumption in the newly added comparison with previous work.

Line 357ff, we added: "*We did not include energy consumption in our model in order to keep it as generic as possible. Depending on the type of network, different mechanisms of energy sources, energy distribution and consumption are possible. As an example, energy could either be "harvested" from the environment by the growing processes, or distributed throughout the network from cell bodies. Other existing models either omit energy consumption as well, or contain only specific energy-related mechanisms such as competition between growth cones or diffusion of proteins from the soma.*"

R4-Q7: For what purpose is table 4 in the article? This is purely a description of Python data types. I think this table needs to be dropped.

Response: Thank you for this suggestion, we dropped Table 4 and merged any relevant information into Table 3. This table now describes not only the relevant attributes of cones, nodes and edges, but also how the actions of a cone change, update or generate the class objects.

Changes to the manuscript: Table 4 was removed, and Table 3 was modified to include some additional information from previous Table 4.

R4-Q8: Authors used the model to simulate the development of two biological network systems: the osteocyte lacuno-canalicular system in bone and the sensory nervous system in Zebrafish. But they obtained only qualitative conclusions that states, that there is growth, and the images obtained are similar to experimental pictures. I believe that it is necessary to provide some quantitative characteristics that would allow readers to estimate how close the simulation results are to experimental results (for example, maybe by using the metrics of random graphs theory).

Response: We completely agree, the demonstrations in the old Figure 6 of the initial version of the manuscript were purely qualitative and insufficient for estimating how close the simulation results are to real networks, as noted also by the other reviewers. We now include a new Figure 6 with quantitative studies where we generated a larger number of random networks for each parameter set. In addition, we performed a systematic study of how variations of local growth parameters impact global topology using random graph metric, following your suggestion. Please also see our response to the questions of reviewer #2, and to question R3-Q1 by reviewer #3.

Changes to the manuscript: Figure 6 was replaced by a new Figure with more quantitative results. The results section referring to Figure 6 was completely rewritten to reflect the new results.

R4-Q9: There are various models that describe growth in biological systems and networks. Authors should provide a brief comparison of their model with existing models, and should specifically indicate the advantages of their model over others.

Response: Thank you for this suggestion – we previously only implicitly mentioned limitations of previous models in the introduction. Instead, we now carry out a systematic comparison

with previous models in the discussion and added a new Supplementary Table 1 listing advantages and differences of our model compared to previously published models.

Changes to the manuscript: A new Supplementary Table was added with a systematic comparison of our model with previous published work. We also extended the paragraph on the discussion comparing our work with previous models (Line 330ff.) and refer to this table.

R4-Q10: In «Conclusions» (line 327) a reference to a specific paper(s) must be given.

Response: We assume that this remark refers to the phrase "real image data", and that the source of the data should be cited. Actually, the image data for anisotropic oriented bone tissue used in our simulations of the osteocyte network were synthetically generated, but real images could be used as well. We modified this sentence to avoid confusion.

Changes to the manuscript: Line 410 now reads: *"…could be* obtained from real image data.*"*
